# Association between Sarcopenic Obesity, Type 2 Diabetes, and Hypertension in Overweight and Obese Treatment-Seeking Adult Women

**DOI:** 10.3390/jcdd5040051

**Published:** 2018-10-20

**Authors:** Dima Kreidieh, Leila Itani, Dana El Masri, Hana Tannir, Roberto Citarella, Marwan El Ghoch

**Affiliations:** 1 Department of Nutrition and Dietetics, Faculty of Health Sciences, Beirut Arab University, P.O. Box 11-5020 Riad El Solh, Beirut 11072809, Lebanon; d.kraydeyeh@bau.edu.lb (D.K.); l.itani@bau.edu.lb (L.I.); dana.masri@bau.edu.lb (D.E.M.); hana.tannir@bau.edu.lb (H.T.); 2 CTR Centre of Rehabilitation Therapy, 42124 Reggio Emilia, Italy; citarella@ctr-re.it; 3 Nutrition and Dietetics Program–CTR Centre, 42124 Reggio Emilia, Italy

**Keywords:** body composition, obesity, sarcopenic obesity, type 2 diabetes, hypertension, metabolic syndrome

## Abstract

The last decade has seen a new condition that describes the coexistence of obesity and sarcopenia, termed sarcopenic obesity (SO). We aimed to assess the prevalence of SO in overweight and obese treatment-seeking adult women and the association with type 2 diabetes, hypertension, and dyslipidemia. A body composition assessment was conducted with an InBody bioimpedance analyser in 154 overweight and obese women referred to the Outpatient Clinic in the Department of Nutrition and Dietetics at Beirut Arab University (BAU) in Lebanon, and 30 normal-weight participants of similar age. The overweight and obese patients were then categorized as being with or without sarcopenia. Thirty-one out of the 154 overweight or obese participants met the criteria for SO and displayed a significantly higher prevalence of type 2 diabetes and hypertension than those without SO. Logistic regression analysis showed that SO increases the odds of having type 2 diabetes and hypertension by nearly 550% (odds ratio = 5.42, 95% confidence interval = 1.37–21.40, *p* < 0.05) after adjusting for central fat, eating habits, level of physical activity, and smoking. SO affects nearly 20% of treatment-seeking overweight and obese adult women. Moreover, SO seems to be strongly associated with type 2 diabetes and hypertension.

## 1. Introduction 

The term sarcopenia was initially used to refer to the age-related progressive loss of lean mass and muscle strength, and is associated with chronic diseases and inflammation, and ultimately an increased mortality rate [1]. Obesity is a condition defined as an excessive accumulation of fat in adipose tissue, and is similarly associated with an increased risk of chronic diseases, disability, and mortality [2]. The coexistence of both conditions is referred to as sarcopenic obesity (SO), with recent studies reporting that these conditions, when combined, may synergistically increase their effects on metabolic disorders, cardiovascular diseases, and mortality [3]. Therefore, identifying SO in patients with obesity is critically important in order to provide targeted interventions [4].

The definition of SO has traditionally relied on body composition phenotypes (i.e., lean mass) [5,6,7,8]. However, modern definitions are based on clinical determinations of low muscle strength (i.e., weak handgrip strength) and poor physical performance (e.g., slow walking) [6,7,8]. Definitions of sarcopenia in obesity, based only on lean mass and physical fitness, without accounting for body mass, may be strongly skewed for at least two reasons. Firstly, patients with obesity tend to have a relatively large lean mass. Hence, sarcopenia criteria based on this parameter may not be met in these individuals in whom the prevalence of sarcopenia may be greatly underestimated [9]. Secondly, low physical fitness is more strongly associated with obesity than with sarcopenia [10].

It has been hypothesized that SO may have negative effects on health [5]. However research has produced inconsistent evidence. Whereas some studies have reported a significant association between SO and cardio-metabolic risk (i.e., hyperglycemia, hypertension, dyslipidemia and insulin resistance), other have shown the opposite [5].

These considerations prompted us to assess the prevalence of sarcopenia in overweight and obese treatment-seeking adult women using a new definition proposed by the Foundation for the National Institutes of Health (FNIH) Sarcopenia Project. This definition, in addition to the appendicular lean mass (ALM), also involves the body mass index (BMI), and seems to suit overweight and obese patients [11]. In order to extend the scope of this study, we set out to examine any potential association between SO and cardio-metabolic diseases, namely type 2 diabetes, hypertension, and dyslipidemia, in this population.

## 2. Experimental Methods 

A total of 154 overweight or obese female patients seeking weight-loss treatment were recruited from consecutive referrals by family doctors to the Nutritional and Weight Management Outpatient Clinic in the Department of Nutrition and Dietetics of Beirut Arab University (BAU) in Lebanon during the period of 2014–2016. Patients were eligible for this study if they were female, aged ≥ 18 years, with a BMI ≥ 25.0 kg/m^2^ and at least one weight loss-responsive comorbidity (i.e., type 2 diabetes, cardiovascular disease, sleep apnea, severe joint disease, or two or more risk factors), as defined by the Adult Treatment Panel III [12]. Patients were excluded if they were male, pregnant or lactating, taking medication that affects body weight, or presenting with medical co-morbidities associated with weight loss, or severe psychiatric disorders. Thirty normal-weight female participants with a 18.5 ≥ BMI < 25 kg/m^2^ were recruited from the general population in various community settings as a comparison group, through a simple randomized community email-based survey sent to members of the BAU and other mailing lists. The study design was reviewed and approved by the Institutional Review Board of BAU, with all participants providing informed consent in writing.

A questionnaire was administered to both test participants and controls in order to retrieve information regarding their medical history, lifestyle (eating habits, levels of physical activity, and smoking), and demographic and social conditions (age, sex, and marital status).

Body weight was measured to the nearest 0.1 kg in the clinical sample using an electronic weighing scale (SECA 2730-ASTRA, Germany). Height was measured to the nearest 0.5 cm using a stadiometer. The BMI of each participant was then determined according to the standard formula of body weight (kg) divided by height (m) squared.

Body composition was measured using a body composition analyser (InBody 170, BIOSPACE, China). This single-frequency device uses eight polar electrodes and a single-point load-cell weighing system in the scale platform and provides separate body mass readings for different segments of the body, and uses an algorithm incorporating impedance, age, and height to estimate total and regional body fat and fat-free mass. The total fat and lean mass percentages and the ALM were calculated using standard formulas [13]. SO was defined using a new definition proposed by the Foundation for the National Institutes of Health (FNIH) Sarcopenia Project; this definition, in addition to the appendicular lean mass (ALM), also involves BMI, and seems to suit overweight and obese patients when the ALM/BMI value is less than 0.512 [11].

Cardio-metabolic disease in this study indicates the presence of any metabolic diseases based on self-reported diagnosis (type 2 diabetes, hypertension, and dyslipidemia), either simultaneously or separately.

Descriptive statistics were calculated as means, standard deviation, frequencies, and proportions. The Chi-squared (*X^2^*) test and t-test were used to compare proportions and means, respectively, between participants with normal weight and those who were overweight and obese, and between participants with and without SO in the clinical sample. Simple and multiple logistic regression analyses were performed to calculate the odds of the presence of cardio-metabolic diseases in the clinical sample with SO. All analyses were performed using SPSS version 25 (IBM Corp.; released 2017; IBM SPSS Statistics for Windows, version 25.0. IBM Corp: Armonk, NY, USA). Statistical significance was considered as *p* < 0.05.

## 3. Results 

The study sample included 184 women: 154 overweight or obese patients (mean BMI of 31.42 ± 4.94 kg/m^2^) and 30 normal weight participants (mean BMI of 22.78 ± 1.67 kg/m^2^). The former group displayed a reduced ALM/BMI ratio (0.59 ± 0.10 vs. 0.73 ± 0.11; *p* < 0.001), whereas the two groups were similar in age (33.26 ± 14.65 vs. 30.03 ± 10.20 years; *p =* 0.149) (Table 1).

According to the definition of SO—ALM divided by BMI minus 0.512 [11]—in the overweight and obesity groups, 31 patients (20.1%) had SO and 123 (79.9%) did not. None of the participants in the normal-weight group was affected by SO (Table 1).

The group with SO, when compared with the group without SO, had a significantly higher BMI, waist-to-hip ratio, total body fat percentage, visceral fat mass, lower total body water, and fat-free mass percentage (Table 2). Moreover, the SO group had a higher prevalence of type 2 diabetes and hypertension (25.8% vs. 8.1%; *p =* 0.006).

Logistic regression analysis showed that having SO increases the odds of having type 2 diabetes and hypertension by nearly 400% (odds ratio (OR) = 3.93, 95% confidence interval (CI) = 1.40–11.03), with the odds increasing to 660% (OR = 6.61, 95% CI = 1.73–25.30) when adjusting for a sedentary lifestyle, fast-food consumption, and smoking, and nearly 550% (OR = 5.42, 95% CI = 1.37–21.40) after adding visceral fat mass to the model (Table 3).

## 4. Discussion

The present study aimed to provide benchmark data on the prevalence of sarcopenia in treatment-seeking overweight and obese adult women to assess any potential association between SO with cardio-metabolic diseases, namely type 2 diabetes, hypertension, and dyslipidemia, in this population. Two major findings were revealed.

Firstly, our assessment of the prevalence of sarcopenia among overweight and obese treatment-seeking women using the new criteria, in line with the definition proposed by the FNIH Sarcopenia Project, revealed a prevalence of SO of 20.1%. This falls within the admittedly large prevalence range of 0 to 85% reported for women [14], depending on the SO definition applied. Higher prevalence tends to be reported in studies accounting for body mass (i.e., BMI), whereas lower prevalence is reported in those that did not [9]. A low prevalence may also be explained by the use of definitions that have primarily been developed from studies on older cohorts, and these may not be applicable to younger adults [9].

Second, nearly 25% of participants with SO had type 2 diabetes or blood hypertension, with both conditions strongly associated. The presence of SO increases the odds of having type 2 diabetes or hypertension by more than 550% after adjusting for lifestyle factors (i.e., sedentary lifestyle, fast-food consumption, and smoking) and central adiposity, which are known to be associated with cardio-metabolic diseases. Our findings are in line with several cross-sectional studies demonstrating that individuals with SO have a high cardio-metabolic risk (i.e., of hyperglycemia, hypertension, dyslipidemia, and insulin resistance) [5]. However, the cross-sectional design, at best, reveals only simple associations between SO and some health parameters, but provides no solid information regarding any causal relationships between the two conditions [15,16].

The clinical implications of our findings are that awareness of the presence of sarcopenia in the obese population should be increased among both clinicians and patients. Secondly, our results reveal the importance of screening for SO in overweight and obese treatment-seeking, since this condition seems to be strongly associated with type 2 diabetes and hypertension.

Our study has certain strengths. To the best of our knowledge, this is the first study to assess SO in the Arab region and one of the few studies to assess sarcopenia among overweight and obese individuals by considering ALM and BMI, a new criterion, and cut-off points proposed by the FNIH Sarcopenia Project [11]. In fact, only one study has used this definition in order to identify SO in Italian treatment-seeking patients with obesity [17].

However, the study has certain limitations. First, our results need to be interpreted with caution because they may not apply to patients treated in other settings (i.e., inpatients, pharmacotherapy, or bariatric surgery). Second, we assessed body composition using an impedance analyser; despite being validated, this method has not yet been accepted as a gold-standard technique for overweight and obese patients [18], especially in terms of lean body mass assessment and regional pattern measurements. Third, no biochemical testing was conducted. This means that we were unable to determine the mechanisms and implications of the sarcopenia we observed in overweight and obese adult women, especially if SO was found to be associated with altered inflammatory biomarkers (i.e., C-reactive protein) [19], which is known to play an important role in cardiovascular disease (i.e., myocardial infarction, stroke, peripheral vascular disease, coronary heart disease, and ischemic heart disease) [20]. Fourth, by studying an exclusively female sample, our findings cannot be extended to men with obesity. Fifth, the cross-sectional design of our study should be considered another limitation. Finally, although we did assess eating habits and levels of physical activity, we relied upon self-reporting and did not objectively measure the assessment of these variables.

## 5. Conclusions

Our findings provide preliminary evidence that nearly 20% of overweight and obese adult women, who are seeking weight-loss treatment, have sarcopenia. This condition seems to be strongly associated with having weight-related diseases such as type 2 diabetes and blood hypertension. Therefore, it is clinically useful to screen SO in this population in order to identify potential treatment strategies (e.g., physical activity interventions, high-protein diets, protein supplements, etc.).

## Figures and Tables

**Table 1:** Socio-demographic, lifestyle, and health characteristics of the study population.

	No. (*n =* 184)	Normal *n =* 30	Overweight-Obesity *n =* 154	Significance	Overweight-Obese *n =* 154		Significance
					Non SO	SO	
**Lifestyle**							
Smoking				X^2^ = 0.01; *p =* 0.92			X^2^ = 0.01; *p =* 0.97
Non-smoker	112 (60.9)	18 (60.0)	94 (61.0)		75 (61.0)	19 (10.3)	
Smoker	72 (39.1)	12 (40.0)	60 (39.0)		48 (39.0)	12 (38.1)	
Physical activity				X^2^ = 0.03; *p =* 0.95			X^2^ = 1.23; *p =* 0.27
Sedentary	154 (83.7)	25 (83.3)	129 (83.8)		101 (82.1)	28 (90.3)	
Active	30 (16.3)	5 (16.7)	25 (16.2)		22 (17.9)	3 (9.7)	
Eating away from home				X^2^ = 0.02; *p =* 0.88			X^2^ = 0.03; *p =* 0.98
No	45 (24.5)	7 (23.3)	38 (24.7)		30 (24.4)	8 (25.8)	
Yes	139 (75.5)	23 (76.6)	116 (75.3)		93 (75.6)	23 (74.2)	
**Disease**							
Type 2 Diabetes				X^2^ = 1.23; *p =* 0.25			X^2^ = 6.25; *p* =0.01
No	175 (96.2)	28 (100.0)	147 (95.5)		120 (97.6)	27 (87.1)	
Yes	7 (3.8)	0 (0.0)	7 (4.5)		3 (2.4)	4 (12.9)	
Hypertension				X^2^ = 3.19; *p =* 0.07			X^2^ = 6.20; *p =* 0.013
No	166 (90.2)	28 (100.0)	138 (89.6)		114 (92.7)	24 (77.4)	
Yes	16 (8.7)	0 (0.0)	16 (10.4)		9 (7.3)	7 (22.6)	
Dyslipidemia				X^2^ = 1.12; *p =* 0.29			X^2^ = 1.80; *p =* 0.179
No	166 (90.2)	27 (96.4)	139 (90.3)		113 (91.9)	26 (83.9)	
Yes	16 (8.7)	1 (3.6)	15 (9.7)		10 (8.1)	5 (16.1)	
Diabetes and/or Hypertension				X^2^ = 3.63; *p =* 0.06			X^2^ = 7.50; *p =* 0.006
No	164 (90.1)	28 (100.0)	136 (88.3)		113 (91.9)	23 (74.2)	
Yes	18 (9.9)	0 (0.0)	18 (11.7)		10 (8.1)	8 (25.8)	
Cardio-metabolic disease				X^2^ = 2.89; *p =* 0.09			X^2^ = 5.34; *p =* 0.021
No	157 (86.3)	27 (96.4)	130 (84.4)		108 (87.8)	22 (71.0)	
Yes	25 (13.7)	1 (3.6)	24 (15.6)		15 (12.2)	9 (29.0)	

**Table 2:** Anthropometric characteristics and body composition patterns in normal-weight participants and overweight and obese patients, categorized with or without sarcopenic obesity.

					Overweight Obesity		
	Total *n =* 184	Normal Weight *n =* 30	Overweight-Obese *n =* 154	Significance	Non SO *n =* 123	SO *n =* 31	Significance
BMI	30.01 ± 5.57	22.78 ± 1.67	31.42 ± 4.94	<0.001	30.18 ± 4.03	36.31 ± 5.23	<0.001
Waist to hip ratio	0.94 ± 0.06	0.86 ± 0.03	0.95 ± 0.05	<0.001	0.94 ± 0.04	0.99 ± 0.04	<0.001
FM	31.84 ± 10.10	19.15 ± 4.23	34.32 ± 8.99	<0.001	32.40 ± 7.88	41.91 ± 9.23	<0.001
FM percentage	41.13 ± 6.93	31.97 ± 5.84	42.91 ± 5.60	<0.001	41.24 ± 4.72	49.54 ± 3.53	<0.001
FFM	43.81 ± 6.20	40.44 ± 3.93	44.46 ± 6.35	<0.001	45.10 ± 6.40	41.95 ± 5.57	0.009
FFM percentage	58.63 ± 7.67	68.04 ± 5.85	56.79 ± 6.57	<0.001	58.39 ± 6.19	50.47 ± 3.54	<0.001
Visceral fat mass	10.99 ± 2.86	7.27 ± 1.74	11.71 ± 2.44	<0.001	11.44 ± 2.37	12.81 ± 2.43	0.007
Total body water	32.03 ± 4.78	29.60 ± 2.88	32.51 ± 4.64	<0.001	32.93 ± 5.04	30.81 ± 4.14	0.018
ALM	17.99 ± 2.93	16.37 ± 2.00	18.31 ± 2.99	<0.001	18.74 ± 2.96	16.60 ± 2.47	<0.001
ALM/BMI	0.61 ± 0.12	0.72 ± 0.11	0.59 ± 0.10	<0.001	0.62 ± 0.09	0.46 ± 0.05	<0.001
Non SO	153 (83.2)	30 (100.0)	123 (79.9)	X^2^ = 7.26, *p =* 0.007			
SO	31 (16.8)	0 (0.0)	31 (20.1)				

BMI: Body Mass Index; FM: Total body fat mass; FFM: Fat Free Mass; ALM: Appendicular lean mass; SO: Sarcopenic Obesity.

**Table 3:** Odds of cardio-metabolic disease with sarcopenic obesity.

	Model 1	Model 2	Model 3
Disease	OR (95% CI)		
Type 2 Diabetes (T2D)			
Non SO	1	1	1
SO	5.93 (1.25–28.03)	5.90 (1.05–33.22)	5.17 (0.76–35.42)
Hypertension			
Non SO	1	1	1
SO	3.69 (1.25–10.89)	5.39 (1.44–20.19)	4.48 (1.16–17.38)
Dyslipidemia			
Non SO	1	1	1
SO	2.17 (0.69–6.90)	2.58 (0.70–9.59)	2.50 (0.66–9.56)
Type 2 Diabetes and/or Hypertension			
Non SO	1	1	1
SO	3.93 (1.40–11.03)	6.61 (1.73–25.30)	5.42 (1.37–21.40)
Cardio-metabolic disease			
Non SO	1	1	1
SO	2.95 (1.15–7.58)	4.74 (1.39–16.10)	4.00 (1.15–13.86)
